# Supplementary material for: Quality-of-Life Determinants in People with Diabetes Mellitus in Europe
Source: Int J Environ Res Public Health. 2021 Jun 28;18(13):6929. doi: 10.3390/ijerph18136929 (PMC8297329; doi:10.3390/ijerph18136929)
Supplement: Supplementary file 1 [file ijerph-18-06929-s001.zip › ijerph-1222870-supplementary.pdf]

International Journal of
Environmental Research and Public Health

MDPI

Supplementary Materials

The attached supplementary information serves to describe and complement the information on our study population and variables. It adds value to the tables in the main document. For this reason, we ask that it be considered for future publication

Supplementary Table S1. Characteristics of total study population.

Characteristics	*n*	%
Diabetes *		
No	291,822	93.5
Yes	20,350	6.5
Sex		
Woman	161,516	51.7
Man	150,656	48.3
Age		
<40 years	117,350	37.6
40–54 years	80,982	25.9
55–64 years	47,552	15.2
65–74 years	35,764	11.5
>74 years	30,523	9.8
Country of residence		
Spain	22,835	7.3
Bulgaria	6,378	2.0
Czech Republic	6,736	2.2
Denmark	5,718	1.8
Germany	24,154	7.7
Estonia	5,453	1.7
Ireland	9,358	3.0
Greece	8,213	2.6
Belgium	9,111	2.9
France	14,898	4.8
Croatia	5,425	1.7
Italy	24,929	8.0
Cyprus	4,954	1.6
Latvia	7,067	2.3
Lithuania	5,209	1.7
Luxembourg	3,953	1.3
Hungary	5,824	1.9
Malta	4,064	1.3
Netherlands	7,649	2.5
Austria	15,763	5.0
Poland	24,115	7.7
Portugal	18,183	5.8
Romania	16,603	5.3
Slovenia	6,214	2.0
Slovakia	5,489	1.8
Finland	5,388	1.7
Sweden	6,169	2.0
United Kingdom	20,123	6.4
Iceland	4,029	1.3
Norway	8,164	2.6
Country of birth		
Native-born	279,959	89.7
Born in another country	29,758	9.5
Don't know/refusal	2,455	0.8
Degree of urbanization		
Densely-populated area	117,966	37.8
Intermediate-populated area	93,636	30.0
Thinly-populated area	100,058	32.1
Don't know/refusal	512	0.2
Legal marital status		

**Characteristics**	***n***	**%**
Never married, never in a registered partnership	98,160	31.4
Married/in a registered partnership	165,800	53.1
Widowed/registered partnership ending in death of partner	25,472	8.2
Divorced/registered partnership that was legally dissolved	22,000	7.0
Don't know/refusal	740	0.2
Educational attainment		
Tertiary education; bachelor, master or doctoral level	58,839	18.8
Tertiary education; short cycle	30,210	9.7
Secondary education	179,868	57.6
Primary education	40,388	12.9
Don't know/refusal	2,867	0.9
Labor status		
In work	155,841	49.9
Unemployed	21,888	7.0
Studying	28,277	9.1
Retired	71,106	22.8
Domestic tasks	16,585	5.3
Other inactive	16,711	5.4
Don't know/refusal	1,763	0.6
Body mass index		
Normal	146,573	47.0
Overweight	104,328	33.4
Obese	46,304	14.8
Don't know/refusal	14,967	4.8
Hours of recreational physical activity		
>7 h a week	15,262	4.9
3 to 7 h a week	36,723	11.8
1 to 3 h a week	67,670	21.7
Don't know/refusal	192,517	61.7
Frequency of eating fruit		
Once or more a day	167,074	53.5
4 to 6 times a week	56,200	18.0
1 to 3 times a week	57,325	18.4
Less than once a week	18,459	5.9
Never	4,645	1.5
Don't know/refusal	8,469	2.7
Frequency of eating vegetables		
Once or more a day	153,613	49.2
4 to 6 times a week	79,421	25.4
1 to 3 times a week	58,213	18.6
Less than once a week	9,971	3.2
Never	2,389	0.8
Don't know/refusal	8,565	2.7
Smoking		
Daily smoker	60,212	19.3
Occasional smoker	15,014	4.8
Non-smoker	232,549	74.5
Don't know/refusal	4,397	1.4
Exposure to smoke		
Never or almost never	230,926	74.0
Less than 1 h a day	33,582	10.8
1 h or more a day	32,423	10.4
Don't know/refusal	15,241	4.9
Alcohol consumption		
Every day or almost every day	19,834	6.4
3 to 6 days a week	23,670	7.6
1 to 2 days a week	109,194	35.0
1 to 3 days a month	100,245	32.1
Less than once a month	0	0.0
Don't know/refusal	59,29	19.0
Number of close people to count on		
None	6,252	2.0

Characteristics	n	%
1 or 2	92,254	29.6
3 to 5	130,937	41.9
6 or more	71,428	22.9
Don't know/refusal	11,301	3.6
Number of people living in household		
One	49,118	15.7
Two	95,350	30.5
Three	66,582	21.3
Four	62,742	20.1
Five or more	38,090	12.2
Don't know/refusal	290	0.1
Type of household		
One-person household	48,987	15.7
Lone parent with child(ren) aged less than 25	14,583	4.7
Couple with child(ren) aged less than 25	95,069	30.5
Couple without child(ren) aged less than 25	82,098	26.3
Other type of household	69,429	22.2
Don't know/refusal	2,006	0.6
Household income		
Below 1st quintile	56,861	18.2
Between 1st and 2nd quintile	56,996	18.3
Between 2nd and 3rd quintile	58,052	18.6
Between 3rd and 4th quintile	59,589	19.1
Between 4th and 5th quintile	60,124	19.3
Don't know/refusal	20,550	6.6
Self-perceived general health		
Very good	74,829	24.0
Good	135,172	43.3
Fair	67,639	21.7
Bad	19,215	6.2
Very bad	4,910	1.6
Don't know/refusal	10,406	3.3
Long-standing health problem (duration ≥ 6 months)		
No	173,215	55.5
Yes	135,017	43.3
Don't know/refusal	3,940	1.3
Asthma *		
No	294,456	94.3
Yes	16,461	5.3
Don't know/refusal	1,254	0.4
COPD *		
No	299,174	95.8
Yes	11,767	3.8
Don't know/refusal	1,231	0.4
MI or chronic consequences of MI *		
No	306,017	98.0
Yes	4,895	1.6
Don't know/refusal	1,260	0.4
Coronary heart disease or angina pectoris *		
No	299,602	96.0
Yes	11,273	3.6
Don't know/refusal	1,297	0.4
Hypertension *		
No	246,317	78.9
Yes	65,340	20.9
Don't know/refusal	516	0.2
Stroke or chronic consequences of stroke *		
No	306,626	98.2
Yes	4,239	1.4
Don't know/refusal	1,307	0.4
Arthrosis (arthritis excluded) *		
No	270,330	86.6

Characteristics	*n*	%
Yes	40,446	13.0
Don't know/refusal	1,396	0.4
Low back disorder or another chronic back defect *		
No	241,379	77.3
Yes	69,659	22.3
Don't know/refusal	1,134	0.4
Neck disorder or other chronic neck defect *		
No	262,375	84.0
Yes	48,667	15.6
Don't know/refusal	1,129	0.4
Allergy (allergic asthma excluded) *		
No	262.954	84.2
Yes	48.580	15.6
Don't know/refusal	637	0.2
Cirrhosis of the liver *		
No	310,301	99.4
Yes	1,221	0.4
Don't know/refusal	649	0.2
Urinary incontinence *		
No	296,329	94.9
Yes	15,269	4.9
Don't know/refusal	574	0.2
Kidney problems *		
No	302,874	97.0
Yes	8,638	2.8
Don't know/refusal	659	0.2
Depression *		
No	289,399	92.7
Yes	21,871	7.0
Don't know/refusal	902	0.3
Road traffic accident *		
No	304,398	97.5
Yes	4,682	1.5
Don't know/refusal	3,092	1.0
Home accident *		
No	298,609	95.7
Yes	10,557	3.4
Don't know/refusal	3,006	1.0
Leisure accident *		
No	296,470	95.0
Yes	12,795	4.1
Don't know/refusal	2,907	0.9
Wearing glasses or contact lenses		
No	132,272	42.4
Yes	179,428	57.5
Don't know/refusal	472	0.2
Use of a hearing aid		
No	300,543	96.3
Yes	10,797	3.5
Don't know/refusal	832	0.3
Physical limitation		
No difficulty	247,192	79.2
Moderate difficulty	28,933	9.3
Severe difficulty	20,371	6.5
Don't know/refusal	15,675	5.0
Intensity of bodily pain during the past 4 weeks		
None	147,610	47.3
Very mild	39,294	12.6
Mild	42,571	13.6
Moderate	46,087	14.8
Severe	21,305	6.8
Very severe	5,951	1.9

Characteristics	*n*	%
Don't know/refusal	9,354	3.0
Depression severity (PHQ-8)		
None/minimal (0–4)	206,477	66.1
Mild (5–9)	36,789	11.8
Moderate (10–14)	9,829	3.1
Severe (15–24)	5,729	1.8
Don't know/refusal	53,349	17.1
Last time of vaccination against flu		
Never or long ago	245,299	78,6
In the past year	49,960	16,0
Don't know/refusal	16,913	5,4

* in the last 12 months; COPD: chronic obstructive pulmonary disease; MI: myocardial infarction.

Supplementary Table S 2. Health status variables in the diabetic and non-diabetic populations.

Characteristics	No diabetes		Diabetes		*P* value
	n	%	n	%	
Sex					
Woman	151,184	51.8	10,331	50.8	0.004
Man	140,638	48.2	10,018	49.2	
Age					
<40 years	116,201	39.8	1,150	5.7	<0.001
40–54 years	77,942	26.7	3,040	14.9	
55–64 years	42,816	14.7	4,737	23.3	
65–74 years	30,046	10.3	5,718	28.1	
>74 years	24,817	8.5	5,705	28.0	
Country of residence					
Spain	21,275	7.3	1,561	7.7	<0.001
Bulgaria	5,972	2.0	406	2.0	
Czech Republic	6,220	2.1	516	2.5	
Denmark	5,454	1.9	264	1.3	
Germany	22,422	7.7	1,732	8.5	
Estonia	5,153	1.8	300	1.5	
Ireland	8,925	3.1	433	2.1	
Greece	7,454	2.6	759	3.7	
Belgium	8,625	3.0	486	2.4	
France	13,411	4.6	1,487	7.3	
Croatia	5,038	1.7	387	1.9	
Italy	23,270	8.0	1,659	8.2	
Cyprus	4,654	1.6	300	1.5	
Latvia	6,738	2.3	329	1.6	
Lithuania	4,980	1.7	230	1.1	
Luxembourg	3,733	1.3	220	1.1	
Hungary	5,354	1.8	469	2.3	
Malta	3,729	1.3	335	1.6	
Netherlands	7,237	2.5	412	2.0	
Austria	14,986	5.1	777	3.8	
Poland	22,515	7.7	1,600	7.9	
Portugal	16,487	5.6	1,696	8.3	
Romania	15,809	5.4	795	3.9	
Slovenia	5,789	2.0	426	2.1	
Slovakia	5,113	1.8	377	1.9	
Finland	4,972	1.7	416	2.0	
Sweden	5,876	2.0	293	1.4	
United Kingdom	18,957	6.5	1,167	5.7	
Iceland	3,857	1.3	172	0.8	
Norway	7,819	2.7	344	1.7	
Country of birth					
Native-born	261,633	89.7	18,325	90.1	0.01
Born in another country	27,917	9.6	1,841	9.0	
Don't know/refusal	2,271	0.8	183	0.9	
Degree of urbanization					

Characteristics	No diabetes		Diabetes		*P* value
	n	%	n	%	
Densely populated area	110,339	37.8	7,627	37.5	0.021
Intermediate-populated area	87,502	30.0	6,134	30.1	
Thinly populated area	93,486	32.0	6,572	32.3	
Don't know/refusal	495	0.2	17	0.1	
Legal marital status					
Never married, never in a registered partnership	95,983	32.9	2,177	10.7	<0.001
Married/in a registered partnership	153,473	52.6	12,327	60.6	
Widowed/regd partnership ending in death of partner	21,293	7.3	4,179	20.5	
Divorced/regd partnership that was legally dissolved	20,397	7.0	1,603	7.9	
Don't know/refusal	676	0.2	64	0.3	
Educational attainment					
Tertiary education; bachelor/master/doctorate	56,770	19.5	2,069	10.2	<0.001
Tertiary education; short cycle	28,946	9.9	1,263	6.2	
Secondary education	169,197	58.0	10,671	52.4	
Primary education	34,224	11.7	6,164	30.3	
Don't know/refusal	2,685	0.9	182	0.9	
Labor status					
In work	151,350	51.9	4,492	22.1	<0.001
Unemployed	20,970	7.2	919	4.5	
Studying	28,045	9.6	231	1.1	
Retired	59,524	20.4	11,582	56.9	
Domestic tasks	15,215	5.2	1,370	6.7	
Other inactive	15,097	5.2	1,615	7.9	
Don't know/refusal	1,622	0.6	141	0.7	
Body mass index					
Normal	142,015	48.7	4,558	22.4	<0.001
Overweight	96,615	33.1	7,713	37.9	
Obese	39,188	13.4	7,115	35.0	
Don't know/refusal	14,004	4.8	963	4.7	
Hours of recreational physical activity					
>7 h a week	14,714	5.0	548	2.7	<0.001
3 to 7 h a week	35,404	12.1	1,319	6.5	
1 to 3 h a week	64,874	22.2	2,796	13.7	
Don't know/refusal	176,830	60.6	15,687	77.1	
Frequency of eating fruit					
Once or more a day	154,356	52.9	1,2718	62,5	<0.001
4 to 6 times a week	53,241	18.2	2,959	14.5	
1 to 3 times a week	54,377	18.6	2,948	14.5	
Less than once a week	17,435	6.0	1,024	5.0	
Never	4,394	1.5	251	1.2	
Don't know/refusal	8,020	2.7	449	2.2	
Frequency of eating vegetables					
Once or more a day	142,888	49.0	10,725	52.7	<0.001
4 to 6 times a week	74,815	25.6	4,606	22.6	
1 to 3 times a week	54,511	18.7	3702	18.2	
Less than once a week	9,280	3.2	691	3.4	
Never	2,242	0.8	146	0.7	
Don't know/refusal	8,085	2.8	480	2.4	
Smoking					
Daily smoker	57,416	19.7	2,797	13.7	<0.001
Occasional smoker	14,493	5.0	521	2.6	
Non-smoker	215,829	74.0	16,720	82.2	
Don't know/refusal	4,084	1.4	312	1.5	
Exposure to smoke					
Never or almost never	214,900	73.6	16,026	78.8	<0.001
Less than 1 h a day	32,054	11.0	1,527	7.5	
1 h or more a day	30,590	10.5	1,833	9.0	
Don't know/refusal	14,278	4.9	963	4.7	
Alcohol consumption					
Every day or almost every day	18,126	6.2	1,708	8.4	<0.001
3 to 6 days a week	22,577	7.7	1,094	5.4	

Characteristics	No diabetes		Diabetes		P value
	n	%	n	%	
1 or 2 days a week	104,606	35.8	4,587	22.5	
1 to 3 days a month	91,530	31.4	8,715	42.8	
Don’t know/refusal	54,983	18.8	4,246	20.9	
Number of close people to count on					
None	5,614	1.9	638	3.1	<0.001
1 or 2	84,626	29.0	7,628	37.5	
3 to 5	123,221	42.2	7,716	37.9	
6 or more	67,702	23.2	3,726	18.3	
Don’t know/refusal	10,659	3.7	642	3.2	
Number of people living in household					
One	44,217	15.2	4,900	24.1	<0.001
Two	86,198	29.5	9,151	45.0	
Three	63,554	21.8	3,027	14.9	
Four	60,905	20.9	1,837	9.0	
Five or more	36,686	12.6	1,405	6.9	
Don’t know/refusal	261	0.1	29	0.1	
Type of household					
One-person household	44,100	15.1	4,887	24.0	<0.001
Lone parent with child(ren) aged less than 25	14,222	4.9	361	1.8	
Couple with child(ren) aged less than 25	92,967	31.9	2,102	10.3	
Couple without child(ren) aged less than 25	73,682	25.2	8,416	41.4	
Other type of household	64,974	22.3	4,454	21.9	
Don’t know/refusal	1,876	0.6	130	0.6	
Household income					
Below 1st quintile	52,710	18.1	4,151	20.4	<0.001
Between 1st and 2nd Quintile	52,125	17.9	4,870	23.9	
Between 2nd and 3rd quintile	53,982	18.5	4,071	20.0	
Between 3rd and 4th quintile	56,249	19.3	3,340	16.4	
Between 4th and 5th quintile	57,464	19.7	2,659	13.1	
Don’t know/refusal	19,292	6.6	1,258	6.2	
Self-perceived general health					
Very good	74,216	25.4	613	3.0	<0.001
Good	130,289	44.6	4,884	24.0	
Fair	58,745	20.1	8,894	43.7	
Bad	15,020	5.1	4,194	20.6	
Very bad	3,707	1.3	1,203	5.9	
Don’t know/refusal	9,844	3.4	562	2.8	
Long-standing health problem (duration ≥6 months)					
No	171,603	58.8	1,612	7.9	<0.001
Yes	116,518	39.9	18,499	90.9	
Don’t know/refusal	3,700	1.3	240	1.2	
Asthma *					
No	277,013	94.9	17,443	85.7	<0.001
Yes	13,986	4.8	2,475	12.2	
Don’t know/refusal	823	0.3	431	2.1	
COPD *					
No	281,369	96.4	17,805	87.5	<0.01
Yes	9,659	3.3	2,108	10.4	
Don’t know/refusal	794	0.3	437	2.1	
MI or chronic consequences of MI *					
No	287,497	98.5	18,520	91.0	<0.001
Yes	3,521	1.2	1,374	6.8	
Don’t know/refusal	804	0.3	457	2.2	
Coronary heart disease or angina pectoris *					
No	282,345	96.8	17,257	84.8	<0.001
Yes	8,616	3.0	2,657	13.1	
Don’t know/refusal	861	0.3	436	2.1	
Hypertension *					
No	238,080	81.6	8,237	40.5	<0.001
Yes	53.366	18.3	11,973	58.8	
Don’t know/refusal	376	0.1	140	0–7	

Characteristics	No diabetes		Diabetes		P value
	n	%	n	%	
Stroke or chronic consequences of stroke *					
No	287,724	98.6	18,902	92.9	<0.001
Yes	3,257	1.1	982	4.8	
Don't know/refusal	841	0.3	466	2.3	
Arthrosis (arthritis excluded) *					
No	256,939	88.0	13,391	65.8	<0.001
Yes	33,890	11.6	6,556	32.2	
Don't know/refusal	993	0.3	403	2.0	
Low back disorder or other chronic back defect *					
No	229,183	78.5	12,195	59.9	<0.001
Yes	61,817	21.2	7,842	38.5	
Don't know/refusal	821	0.3	312	1.5	
Neck disorder or other chronic neck defect *					
No	247,808	84.9	14,567	71.6	<0.001
Yes	43,275	14.8	5,392	26.5	
Don't know/refusal	738	0.3	391	1.9	
Allergy (allergic asthma excluded) *					
No	246,278	84.4	16,677	82.0	<0.001
Yes	45,277	15.5	3,303	16.2	
Don't know/refusal	267	0.1	370	1.8	
Cirrhosis of the liver *					
No	290,731	99.6	19,570	96.2	<0.001
Yes	869	0.3	353	1.7	
Don't know/refusal	222	0.1	427	2.1	
Urinary incontinence *					
No	279,400	95.7	16,929	83.2	<0.001
Yes	12,202	4.2	3,067	15.1	
Don't know/refusal	220	0.1	354	1.7	
Kidney problems *					
No	284,834	97.6	18,040	88.6	<0.001
Yes	6,726	2.3	1,912	9.4	
Don't know/refusal	262	0.1	398	2.0	
Depression *					
No	272,173	93.3	17,226	84.6	<0.001
Yes	19,134	6.6	2,738	13.5	
Don't know/refusal	516	0.2	386	1.9	
Road traffic accident					
No	284,639	97.5	19,759	97.1	<0.001
Yes	4,425	1.5	257	1.3	
Don't know/refusal	2,758	0.9	334	1.6	
Home accident *					
No	279,777	95.9	18,832	92.5	<0.001
Yes	9,348	3.2	1,209	5.9	
Don't know/refusal	2,697	0.9	309	1.5	
Leisure accident *					
No	277,050	94.9	19,419	95.4	<0.001
Yes	12,211	4.2	584	2.9	
Don't know/refusal	2,560	0.9	346	1.7	
Wearing glasses or contact lenses					
No	128,205	43.9	4,067	20.0	<0.001
Yes	163,187	55.9	16.241	79.8	
Don't know/refusal	430	0.1	42	0.2	
Use of a hearing aid					
No	281,853	96.6	18,690	91.8	<0.001
Yes	9,233	3.2	1,563	7.7	
Don't know/refusal	735	0.3	96	0.5	
Physical limitation					
No difficulty	236,831	81.2	10,362	50.9	<0.001
Moderate difficulty	24,361	8.3	4,572	22.5	
Severe difficulty	15,626	5.4	4,746	23.3	

Characteristics	No diabetes		Diabetes		P value
	n	%	n	%	
Don't know/refusal	15,005	5.1	670	3.3	
Intensity of bodily pain during the past 4 weeks					
None	141,928	48.6	5,682	27.9	<0.001
Very mild	37,087	12.7	2,207	10.8	
Mild	39,509	13.5	3,062	15.0	
Moderate	41,170	14.1	4,916	24.2	
Severe	18,307	6.3	2,998	14.7	
Very severe	4,966	1.7	985	4.8	
Don't know/refusal	8,854	3.0	500	2.5	
Depression severity (PHQ-8)					
None (0–4)	195,611	67.0	10,866	53.4	<0.001
Mild (5–9)	33,189	11.4	3,599	17.7	
Moderate (10–14)	8,451	2.9	1,378	6.8	
Severe (15–24)	4,807	1.6	923	4.5	
Don't know/refusal	49,764	17.1	3,584	17.6	
Last time of vaccination against flu					
Never or long ago	233,812	80.1	11,486	56.4	<0.001
In the past year	42,083	14.4	7,877	38.7	
Don't know/refusal	15,927	5.5	987	4.9	

* in the past 12 months; COPD: chronic obstructive pulmonary disease; MI: myocardial infarction.

Supplementary Table S3. Multivariate logistic regression model for the presence of diabetes.

Characteristics	OR	95% CI	P value
Sex			
Woman	1		
Man	1.46	(1.40–1.53)	<0.001
Age			
<40 years	1		
40–54 years	1.92	(1.74–2.11)	<0.001
55–64 years	2.96	(2.67–3.29)	<0.001
65–74 years	3.47	(3.09–3.89)	<0.001
>74 years	3.28	(2.86–3.65)	<0.001
Country of residence			
Bulgaria	1		
Czech Republic	1.34	(1.16–1.56)	<0.001
Denmark	1.61	(1.34–1.93)	<0.001
Germany	1.64	(1.44–1.87)	<0.001
Estonia	0.79	(0.67–0.94)	0.009
Ireland	1.23	(1.04–1.46)	0.018
Greece	1.39	(1.20–1.61)	<0.001
Croatia	1.05	(0.90–1.24)	0.528
Cyprus	1.31	(1.10–1.56)	0.002
Latvia	0.69	(0.59–0.82)	<0.001
Lithuania	0.60	(0.50–0.72)	<0.001
Luxembourg	1.93	(1.59–2.36)	<0.001
Hungary	1.29	(1.10–1.51)	0.001
Malta	1.90	(1.59–2.29)	<0.001
Austria	1.67	(1.44–1.92)	<0.001
Poland	1.06	(0.94–1.20)	0.351
Portugal	1.47	(1.28–1.68)	<0.001
Romania	1.18	(1.03–1.36)	0.014
Slovenia	1.61	(1.37–1.89)	<0.001
Slovakia	1.12	(0.96–1.32)	0.148
Finland	1.27	(1.04–1.54)	0.019
Sweden	1.47	(1.23–1.76)	<0.001
United Kingdom	1.16	(1.01–1.33)	0.030
Iceland	1.34	(1.09–1.65)	0.006
Norway	1.71	(1.44–2.02)	<0.001
Country of birth			
Native-born	1		

Characteristics	OR	95% CI	*P* value
Born in another country	1.20	(1.12–1.27)	<0.001
Don't know/refusal	1.39	(0.88–2.16)	0.159
Degree of urbanization			
Densely populated area	1		
Intermediate-populated area	0.96	(0.92–1.01)	0.1022
Thinly populated area	0.88	(0.84–0.93)	<0.001
Don't know/refusal	0.67	(0.40–1.12)	0,124
Marital status			
Never married, never in a registered partnership	1		
Married/in a registered partnership	0.99	(0.92–1.07)	0.884
Widowed/registered partnership ending in death of partner	1.12	(1.03–1.22)	0.009
Divorced/registered partnership that was legally dissolved	0.98	(0.90–1.08)	0.729
Don't know/refusal	1.08	(0.79–1.48)	0.614
Educational attainment			
Tertiary education; bachelor, master or doctoral level	1		
Tertiary education; short cycle	0.98	(0.90–1.08)	0.752
Secondary education	1.14	(1.07–1.22)	<0.001
Primary education	1.29	(1.19–1.41)	<0.001
Don't know/refusal	1.15	(0.88–1.50)	0,306
Labor status			
In work	1		
Unemployed	1.09	(0.90–1.21)	0.088
Studying	0.85	(0.70–1.02)	0.088
Retired	1.21	(1.13–1.30)	<0.001
Domestic tasks	1.28	(1.15–1.43)	<0.001
Other inactive	1.14	(1.05–1.24)	0.001
Don't know/refusal	1.20	(0.97–1.48)	0.094
Body mass index			
Normal	1		
Overweight	1.57	(1.49–1.65)	<0.001
Obese	2.75	(2.60–2.90)	<0.001
Don't know/refusal	1.73	(1.53–1.95)	<0.001
Hours of recreational physical activity			
>7 h/week	1		
3–7 h/week	1.01	(0.89–1.15)	0.821
1 to 3 h a week	1.04	(0.93–1.17)	0.485
Don't know/refusal	1.14	(1.02–1.28)	0.020
Frequency of eating fruit			
Once or more a day	1		
4 to 6 times a week	0.90	(0.85–0.95)	<0.001
1 to 3 times a week	0.86	(0.81–0.91)	<0.001
Less than once a week	0.88	(0.81–0.95)	0.001
Never	0.81	(0.68–0.97)	0.025
Don't know/refusal	0.74	(0.53–1.03)	0.079
Alcohol consumption			
Every day or almost every day	1		
3 to 6 days a week	0.93	(0.85–1.03)	0.156
1 to 2 days a week	1.11	(1.04–1.20)	0.003
1 to 3 days a month	1.42	(1.32–1.52)	<0.001
Don't know/refusal	1.48	(1.20–1.82)	<0.001
Number of close people to count on			
None	1		
1 or 2	0.86	(0.76–0.96)	0.008
3 to 5	0.82	(0.73–0.92)	<0.001
6 or more	0.86	(0.76–0.97)	0.013
Don't know/refusal	0.84	(0.65–1.09)	0.195
Type of household			
One-person household	1		
Lone parent with child(ren) aged less than 25	0.97	(0.84–1.13)	0.732
Couple with child(ren) aged less than 25	0.95	(0.87–1.04)	0.316
Couple without child(ren) aged less than 25	1.09	(1.02–1.17)	0,016
Other type of household	1.05	(0.99–1.12)	0.131

Characteristics	OR	95% CI	*P* value
Don't know/refusal	1.00	(0.80–1.23)	0.967
Household income			
Below 1st quintile	1		
Between 1st and 2nd quintile	1.09	(1.03–1.16)	0.002
Between 2nd and 3rd quintile	1.02	(0.97–1.09)	0.412
Between 3rd and 4th quintile	1.01	(0.95–1.08)	0.744
Between 4th and 5th quintile	1.00	(0.93–1.08)	0.918
Don't know/refusal	0.97	(0.88–1.07)	0.554
Self-perceived general health			
Very good	1		
Good	1.61	(1.45–1.80)	<0.001
Fair	2.52	(2.25–2.81)	<0.001
Bad	3.42	(3.03–3.87)	<0.001
Very bad	3.38	(2.92–3.91)	<0.001
Don't know/refusal	1.88	(1.35–2.60)	<0.001
Long-standing health problem (duration ≥6 months)			
No	1		
Yes	7.39	(6.85–7.97)	<0.001
Don't know/refusal	2.31	(1.66–3.21)	<0.001
Asthma *			
No	1		
Yes	0.88	(0.82–0.94)	<0.001
Don't know/refusal	0.99	(0.69–1.40)	0.937
COPD *			
No	1		
Yes	0.89	(0.82–0.95)	0.001
Don't know/refusal	0.94	(0.65–1.36)	0.735
MI or chronic consequences of MI *			
No	1		
Yes	1.25	(1.15–1.36)	<0.001
Don't know/refusal	1.51	(1.06–2.16)	0.023
Coronary heart disease or angina pectoris *			
No	1		
Yes	1.09	(1.03–1.16)	0.003
Don't know/refusal	1.12	(0.82–1.53)	0.462
Hypertension *			
No	1		
Yes	1.64	(1.58–1.71)	<0.001
Don't know/refusal	1.64	(1.13–2.39)	0.009
Arthrosis (arthritis excluded) *			
No	1		
Yes	0.89	(0.85–0.94)	<0.001
Don't know/refusal	0.97	(0.75–1.26)	0.843
Low back disorder or another chronic back defect *			
No	1		
Yes	0.78	(0.74–0.81)	<0.001
Don't know/refusal	0.72	(0.51–1.01)	0,058
Neck disorder or other chronic neck defect *			
No	1		
Yes	0.91	(0.87–0.96)	<0.001
Don't know/refusal	1.50	(1.08–2.09)	0.016
Allergy (allergic asthma excluded) *			
No	1		
Yes	0.93	(0.88–0.98)	0.009
Don't know/refusal	2.13	(1.51–3.01)	<0.001
Cirrhosis of the liver *			
No	1		
Yes	1.97	(1.68–2.32)	<0.001
Don't know/refusal	2.94	(2.03–4.28)	<0.001
Urinary incontinence *			
No	1		

Characteristics	OR	95% CI	*P* value
Yes	1.11	(1.04–1.18)	<0.001
Don't know/refusal	3.32	(2.31–4.79)	<0.001
Kidney problems *			
No	1		
Yes	1.50	(1.40–1.61)	<0.001
Don't know/refusal	1.565	(1.06–2.30)	0.022
Home accident *			
No			
Yes	1.15	(1.06–1.25)	0.001
Don't know/refusal	0.82	(0.68–0.99)	0.034
Wearing glasses or contact lenses			
No	1		
Yes	1.19	(1.13–1.25)	<0.001
Don't know/refusal	0.78	(0.51–1.21)	0.270
Physical limitation			
No difficulty			
Moderate difficulty	1.10	(1.04–1.15)	<0.001
Severe difficulty	1.19	(1.12–1.27)	<0.001
Don't know/refusal	1.16	(0.83–1.62)	0.372
Intensity of bodily pain during the past 4 weeks			
None	1		
Very mild	0.87	(0.83–0.93)	<0.001
Mild	0.76	(0.71–0.81)	<0.001
Moderate	0.74	(0.70–0.79)	<0.001
Severe	0.70	(0.66–0.76)	<0.001
Very severe	0.77	(0.69–0.86)	<0.001
Don't know/refusal	0.73	(0.54–0.98)	0.039
Last time of vaccination against flu			
Never or long ago	1		
In the past year	1.64	(1.56–1.72)	<0.001
Don't know/refusal	1.37	(1.18–1.59)	<0.001

* in the past 12 months; OR: Odds Ratio; COPD: chronic obstructive pulmonary disease; MI: myocardial infarction.

Supplementary Table S4. Model indicators.

n	Diabetes	LRT	*P* value	AUC	95% CI
232,386	17,029	32,071.1	<0.001	0.87	(0.87–0.88)

LRT: likelihood ratio test; AUC: area under the receiver operating characteristic curve.
